# CXCR4 blockade decreases CD4^+^ T cell exhaustion and improves survival in a murine model of polymicrobial sepsis

**DOI:** 10.1371/journal.pone.0188882

**Published:** 2017-12-12

**Authors:** Kimberly M. Ramonell, Wenxiao Zhang, Annette Hadley, Ching-wen Chen, Katherine T. Fay, John D. Lyons, Nathan J. Klingensmith, Kevin W. McConnell, Craig M. Coopersmith, Mandy L. Ford

**Affiliations:** 1 Department of Surgery, Emory University School of Medicine, Atlanta, Georgia, United States of America; 2 Emory Critical Care Center, Emory University School of Medicine, Atlanta, Georgia, United States of America; 3 Emory Transplant Center, Emory University School of Medicine, Atlanta, Georgia, United States of America; National Cancer Institute, UNITED STATES

## Abstract

Sepsis is a dysregulated systemic response to infection involving many inflammatory pathways and the induction of counter-regulatory anti-inflammatory processes that results in a state of immune incompetence and can lead to multi-organ failure. CXCR4 is a chemokine receptor that, following ligation by CXCL12, directs cells to bone marrow niches and also plays an important role in T cell cosignaling and formation of the immunological synapse. Here, we investigated the expression and function of CXCR4 in a murine model of polymicrobial sepsis. Results indicate that CXCR4 is selectively upregulated on naïve CD4^+^ and CD8^+^ T cells and CD4^+^ central memory T cells following the induction of sepsis, and that CXCR4 antagonism resulted in a significant decrease in sepsis-induced mortality. We probed the mechanistic basis for these findings and found that CXCR4 antagonism significantly increased the number of peripheral CD4^+^ and CD8^+^ T cells following sepsis. Moreover, mice treated with the CXCR4 antagonist contained fewer PD-1^+^ LAG-3^+^ 2B4^+^ cells, suggesting that blockade of CXCR4 mitigates CD4^+^ T cell exhaustion during sepsis. Taken together, these results characterize CXCR4 as an important pathway that modulates immune dysfunction and mortality following sepsis, which may hold promise as a target for future therapeutic intervention in septic patients.

## Introduction

Sepsis is life-threatening organ dysfunction caused by a dysregulated host response to infection and is responsible for more than 300,000 deaths annually [1, 2]. With the exception of antibiotics, current therapy is limited to non-specific supportive care and mortality remains at 40% [3, 4]. However, there is increasing appreciation for the central role that immunologic dysfunction plays in driving sepsis mortality. In particular, the immunosuppressive phase of sepsis contributes to impaired immune competency, susceptibility to secondary infections and increased mortality in septic patients [5–7]. A number of interacting processes contribute to this state, including apoptosis of immune effector cells, expansion of immunosuppressive T regulatory (T_Reg_) cells, T cell exhaustion, and monocyte deactivation [8, 9].

Additionally, sepsis triggers extensive apoptosis-induced depletion of innate and adaptive immune cells and some remaining cells are rendered dysfunctional or exhausted, due to the prolonged exposure to excessive pro- and anti-inflammatory cytokines. Phenotypically, immune cell exhaustion is characterized by increased expression of co-inhibitory markers including programmed cell death (PD-1), 2B4, BTLA, and LAG-3 on CD4^+^ and CD8^+^ T cells. Signaling through these coinhibitory molecules may limit the ability of T cells to proliferate and produce cytokines and attenuate cytotoxic T cell function [10, 11]. For instance, PD-1 overexpression on circulating T cells from septic patients correlates with decreased T cell proliferative capacity, increased secondary nosocomial infections, and increased mortality. Pharmacologic blockade of T cell coinhibitory pathways such as PD-1, BTLA, and 2B4 has been shown to at least partially reverse the state of immune dysregulation and improve survival in pre-clinical models of sepsis [12–19] and PD-1 blockers are currently under investigation for use in clinical sepsis.

Moreover, emerging evidence shows a correlation between lymphopenia and impaired immune cell function, underscoring the importance of restoring both number and function to both innate and adaptive immune systems when treating sepsis [20]. The chemokine receptor CXCR4 and its ligand CXCL12 are involved in regulating the homeostatic recirculation and retention of myeloid and lymphoid cells in the bone marrow [21–25]. CXCR4 is expressed on B and T lymphocytes, dendritic cells, and monocytes [25] and inhibition of CXCR4/CXCL12 signaling results in the release of these cells into the circulation, increasing peripheral absolute cell counts [25]. Interestingly, a recent study of human septic patients revealed that CXCL12 levels were higher in patients with severe sepsis/septic shock as compared to healthy subjects. Moreover, the same study also found that patients who survived their septic insult possessed lower serum levels of CXCL12 than those who died [26]. Thus, we hypothesized that mitigating the detrimental effects of sepsis-induced immune dysfunction by restoring depleted or dysfunctional immune effector cells with functional cells mobilized from bone marrow stores may be beneficial in sepsis. We sought to test this hypothesis by evaluating the effect of CXCR4 blockade on sepsis-induced mortality and immune dysregulation using plerixafor (AMD3100), a CXCR4-antagonist currently FDA approved for stem cell mobilization prior to autologous bone marrow transplantation that is also being investigated as a treatment for several chronic inflammatory diseases including rheumatoid arthritis and inflammatory bowel disease [27–30].

## Materials & methods

### Mice

Adult male and female 9–13 week old C57BL/6 mice were obtained from The Jackson Laboratory (Bar Harbor, ME). All mice were maintained in the same facilities and allowed to acclimate at least one week prior to surgery. Experiments were conducted with approval of the Institutional Animal Care and Use Committee of Emory University (protocol number DAR-2003199-071415N).

### Cecal ligation and puncture (CLP)

Sepsis was induced using CLP, a murine model of polymicrobial sepsis. Injury was titrated to achieve a ~50% 14-day mortality to mimic the clinical scenario of sepsis [31]. In brief, C57BL/6 mice were anesthetized using isoflurane and underwent laparotomy, the cecum was exteriorized, ligated distal to the ileocecal valve, and punctured twice with a 25-gauge needle. Sham-operated animals underwent laparotomy and exteriorization of the cecum only. Personnel conducting sham and CLP surgeries received training and competency testing from Emory University Division of Animal Resources veterinary staff. All animals received buprenorphine (0.1mg/kg) preoperatively for pain relief and 1mL of normal saline for intraoperative fluid losses as well as antibiotics (ceftriaxone 25mg/kg and metronidazole 12.5mg/kg) subcutaneously postoperatively. Antibiotics were continued on a q12hr dosing schedule for 48 hours postoperatively. For experiments evaluating CXCR4 blockade, mice designated to the CLP+ Plerixafor group received a 100μl SQ injection of 5mg/kg Plerixafor (AMD3100, Sigma-Aldrich) one hour after abdominal closure. Throughout this manuscript, mice that underwent CLP and were given normal saline as a control injection are referred to as “septic control mice”. Mice were sacrificed by CO_2_ asphyxiation. For survival studies, mice were observed daily for 7-days after surgery. Animals were observed every 12 hours during this 7-day period. The following criteria were used as humane endpoints; animals meeting any one of these criteria were considered moribund, counted as deceased in the enumeration of surviving animals, and sacrificed by CO_2_ asphyxiation. 1) Loss of 25% of body weight from baseline weight. 2) Major organ failure or medical conditions unresponsive to treatment such as severe respiratory distress, icterus, uremia, intractable diarrhea, or self-mutilation. 3) Surgical complications unresponsive to immediate intervention (bleeding, infection, wound dehiscence). 4) Clinical or behavioral signs unresponsive to appropriate intervention persisting for 24 hours including significant inactivity, labored breathing, sunken eyes, hunched posture, piloerection/matted fur, and abnormal vocalization when handled. Once any animal reached endpoint criteria, the amount of time elapsed before euthanasia was <12 hours. Some animals died before meeting the criteria for euthanasia. A total of 42 animals were used in the survival study, and 26 of them died or met endpoint criteria. The cause of death for all animals was septic shock.

### Immunophenotyping by flow cytometry

Groups of mice were sacrificed at 24 hours following surgery. Spleens were removed aseptically, placed in a 10mL culture dish containing 5mL of PBS (Mediatech, Herndon, VA), and disrupted using the rubber end of a sterile 3mL syringe. Cells were passed through a 70μm mesh filter (BD Falcon), and single cell suspensions were centrifuged and resuspended in 10mL of PBS. Whole blood was harvested by cardiac puncture, 100μL per sample was aliquoted into 12 x 75mm flow tubes and incubated in 2mL of HYL solution (Thermo Fisher Scientific, Waltham, MA, USA) for 15 minutes followed by centrifugation and resuspension in 1mL PBS. For splenic and blood samples, cells were counted by trypan blue exclusion staining, and suspensions adjusted to a concentration of 1x10^7^ cells/mL. Aliquots containing 200uL were apportioned into FACS tubes then centrifuged and resuspended in 100uL FACS Buffer (PBS + 2% FCS + 0.1% Sodium Azide). Samples were pre-incubated with anti-CD16/CD32 mAb (BD Bioscience) for 15m, followed by 30m incubation with the following fluorochrome-conjugated mAbs: CD3 (Biolegend, 17A2), CD4 (BD Biosciences, RM4-5), CD8 (Invitrogen, MCD0830), B220 (BD Biosciences, RA3-6B2), CD44 (BD Biosciences, IM7), CD62L (BD Biosciences, MEL-14), PD-1 (Biolegend, 29F.1A12), LAG-3 (Biolegend, C9B7W), and 2B4 (BD Biosciences, 2B4). Accucheck Counting beads (Invitrogen) were added prior to data collection, per manufacturer’s protocols, and samples were analyzed on a BD LSRII Cytometer. Flow cytometric data were analyzed using FlowJo Software (Treestar, Ashland, OR). Absolute cell counts (per sample) were determined by factoring in the absolute number of cells per spleen enumerated at the time of harvest according to previously published protocols [32].

### Intracellular cytokine staining

2x10^6^ splenocytes from each sample were plated in a 96-well plate. After centrifugation, cells were resuspended and incubated in culture medium (R10) consisting of RPMI 1640 containing 10% FBS (Mediatech, Herndon, VA), 2mM L-glutamine, 0.01 M HEPES buffer, 100mg/ml gentamicin (Mediatech), and 5×10^-5^M 2-mercaptoethanol (Sigma-Aldrich, St. Louis, MO). To test intracellular cytokine, cells were stimulated with 30 mg/ml PMA and 400 ng/ml ionomycin in the presence of GolgiStop (BD Pharmingen) for 4 hours at 37°C.

After incubation and stimulation, cells were surface-stained with anti-CD3-PB (BD), anti-CD4-PerCP (BD), anti-CD8-PO (Biolegend). Then cells were permeabilized using fixation and permeabilization solution (BD). We used anti-IL-2-FITC (BD), anti-TNF-PE-Cy7 (Biolegend) and anti-IFN-γ-Alexa 700 (BD) for intracellular cytokine staining. Samples were analyzed on an LSRII flow cytometer (BD) and data were analyzed using FlowJo software (Tree Star, Ashland, OR, USA).

### Cytokine quantification

Sham, CLP, and CLP + Plerixafor mice were sacrificed at 24h following surgery and whole blood was aspirated via cardiac puncture. Following 30m of incubation, samples were centrifuged (1000g x 10m) and supernatant (serum) was apportioned into 100uL aliquots and stored at -80°C until use. Serum cytokines were evaluated using BioPlex suspension array system and BioPlex Mouse Cytokine 11-Plex Panel according to the manufacturer’s instructions (both Bio-Rad, Marnes-La-Coquette France). Cytokine assays included antibodies for: IL-1β, IL-2, IL-3, IL-4, IL-5, IL-6, IL-10, IL-13, IL-17, IFNγ, MIP-1b, and TNF. Results were analyzed using Bio-Plex Manager^™^ 3.0 software with 5PL curve fitting for determination of serum concentrations (pg/mL) of individual cytokines per sample.

## Statistical analysis

Data were analyzed using the statistical software Prism V; all data are reported as mean +/- SEM. For comparison of absolute cell counts and frequencies of exhausted immune cells across two groups, the Mann Whitney non-parametric test was used. For comparison of three groups, One-way ANOVA and Tukey’s post-test were used. Survival studies were analyzed by Mantel-Cox analysis. For all data, a p-value of ≤ 0.05 was used to determine significance.

## Results

### Sepsis increases the frequency of CXCR4^+^ cells within CD4^+^ T_naive_ and T_CM_ and CD8^+^ T_naive_ subsets

**Fig 1:**
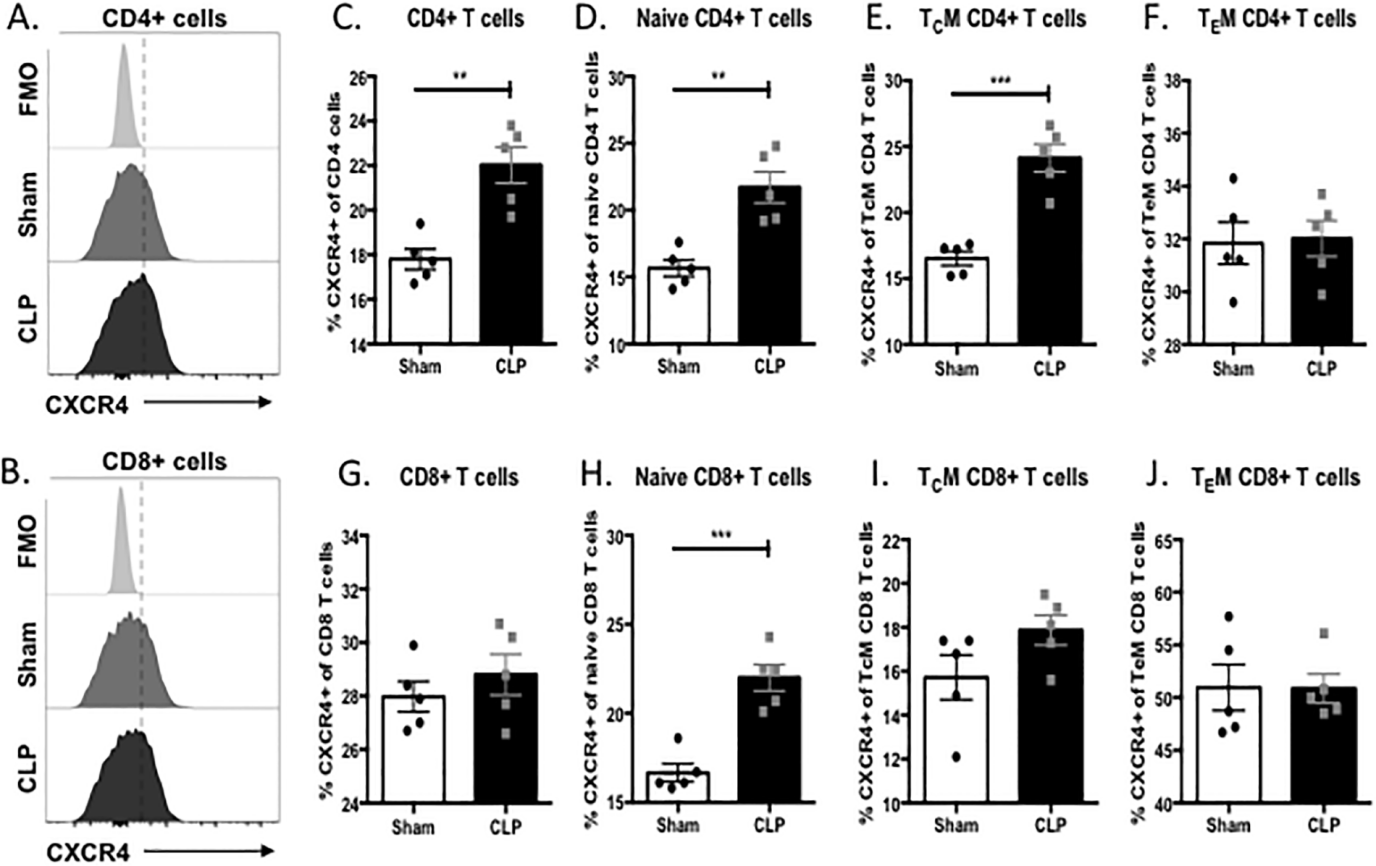
CXCR4 was upregulated on less differentiated T cell subsets during sepsis. Representative histograms demonstrating the expression of CXCR4 on **(A)** CD4^+^ T cells and **(B)** CD8^+^ T cells. **(C)** The frequency of CXCR4 expression was increased in septic mice compared to sham mice on total CD4^+^ T cells in the spleen (22% vs 17.8%; p = 0.002). When the CD4^+^ T cell compartment was further analyzed, the increase in CXCR4 expression on CD4 T cells was limited to (**D**) naïve CD4^+^ T cells (21.7% vs. 15.6%; p = 0.001) and (**E**) central memory CD4^+^ T cells (24.1% vs. 16.5%; p = 0.0002). (**F**) There was no difference in the frequency of CXCR4 expression on effector memory CD4^+^ T cells in septic mice compared to sham mice (32% vs. 31.8%; p = 0.86). (**G**) There was no difference in the frequency of CXCR4^+^ total CD8^+^ T cells in septic mice compared to sham mice (28.8% vs. 27.9%; p = 0.4136). However, the frequency of CXCR4^+^ naïve CD8^+^ T cells (**H**) was significantly increased in septic mice compared to sham mice (22% vs. 16.6%; p = 0.0003) but there were no differences in the frequency of CXCR4^+^ central memory CD8^+^ T cells (**I**; 17.8% vs. 15.7%; p = 0.115) or effector memory CD8^+^ T cells (**J**; 50.8% vs. 50.9%; p = 0.970). Data shown are n = 5/group, representative of a total of 3 independent experiments with a total of n = 15/group.

To determine the expression profile of CXCR4 in the setting of sepsis, mice underwent CLP following by sacrifice and splenic harvest for immunophenotyping of CD4^+^ and CD8^+^ T cells at 24-hours post-sepsis induction (Fig 1A and 1B). The frequency of CXCR4^+^ cells among total CD4^+^ T cells was increased in septic mice compared to sham mice in the spleen (22% vs 17.8%; p = 0.002; Fig 1C). When the CD4^+^ T cell compartment was further analyzed, it was noted that the increase in frequency of CXCR4-expressing CD4^+^ T cells was limited to naïve (CD44^LO^CD62L^HI^) CD4^+^ T cells (21.7% vs. 15.6%; p = 0.001; Fig 1D) and central memory (T_C_M; CD44^HI^CD62L^HI^) CD4^+^ T cells (24.1% vs. 16.5%; p = 0.0002; Fig 1E). In contrast, there was no difference in the frequency of CXCR4-expressing effector memory (T_E_M CD44^HI^CD62L^LO^) CD4^+^ T cells in septic mice compared to sham mice (32% vs. 31.8%; p = 0.86; Fig 1F) suggesting that sepsis induces an upregulation of CXCR4 on less differentiated or antigen experienced CD4^+^ T cells. We then turned our attention toward the CD8^+^ T cell compartment. We found no difference in the frequency of CXCR4^+^ total CD8^+^ T cells in septic mice compared to sham mice (28.8% vs. 27.9%; p = 0.4136; Fig 1G). However, the frequency of CXCR4^+^ naïve CD8^+^ T cells was significantly increased in septic mice compared to sham mice (22% vs. 16.6%; p = 0.0003; Fig 1H) but there were no differences in the frequencies of CXCR4^+^ cells among central memory CD8^+^ T cells (17.8% vs. 15.7%; p = 0.115; Fig 1I) or effector memory CD8^+^ T cells (50.8% vs. 50.9%; p = 0.970; Fig 1J)

### Plerixafor administration improved 7-day survival in murine model of polymicrobial sepsis

**Fig 2:**
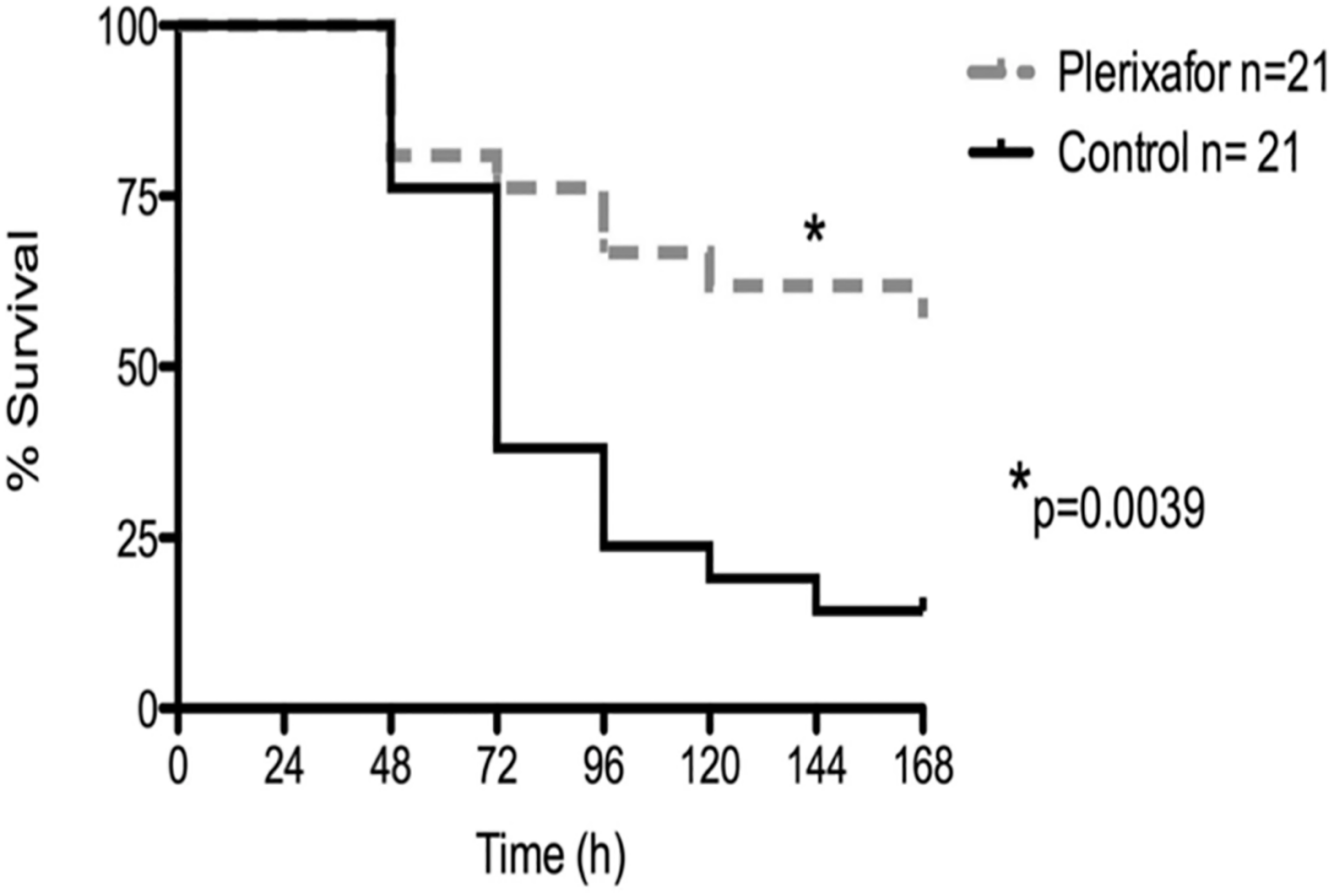
Plerixafor administration improved 7-day survival in murine sepsis. Animals were subjected to CLP and were treated with plerixafor at one hour post-CLP as described in Materials and Methods. Plerixafor administration increased survival from 20% to 65% (n = 21/group; p = 0.0039).

Given the finding that CXCR4 is upregulated on CD4^+^ and CD8^+^ T cells during sepsis, we hypothesized that blocking these signals using a CXCR4 blocking agent could improve survival and immune dysregulation in a murine model of polymicrobial sepsis. To test this, groups of mice were treated with plerixafor as described in materials and methods or were left untreated, and all animals underwent CLP. Results indicated that mice treated with plerixafor one-hour post-sepsis induction had significantly improved 7-day survival compared to septic mice treated with isotype control. Survival was improved from 20% to 65% (Fig 2).

### Plerixafor administration abrogated the loss of peripheral T cells during sepsis

**Fig 3:**
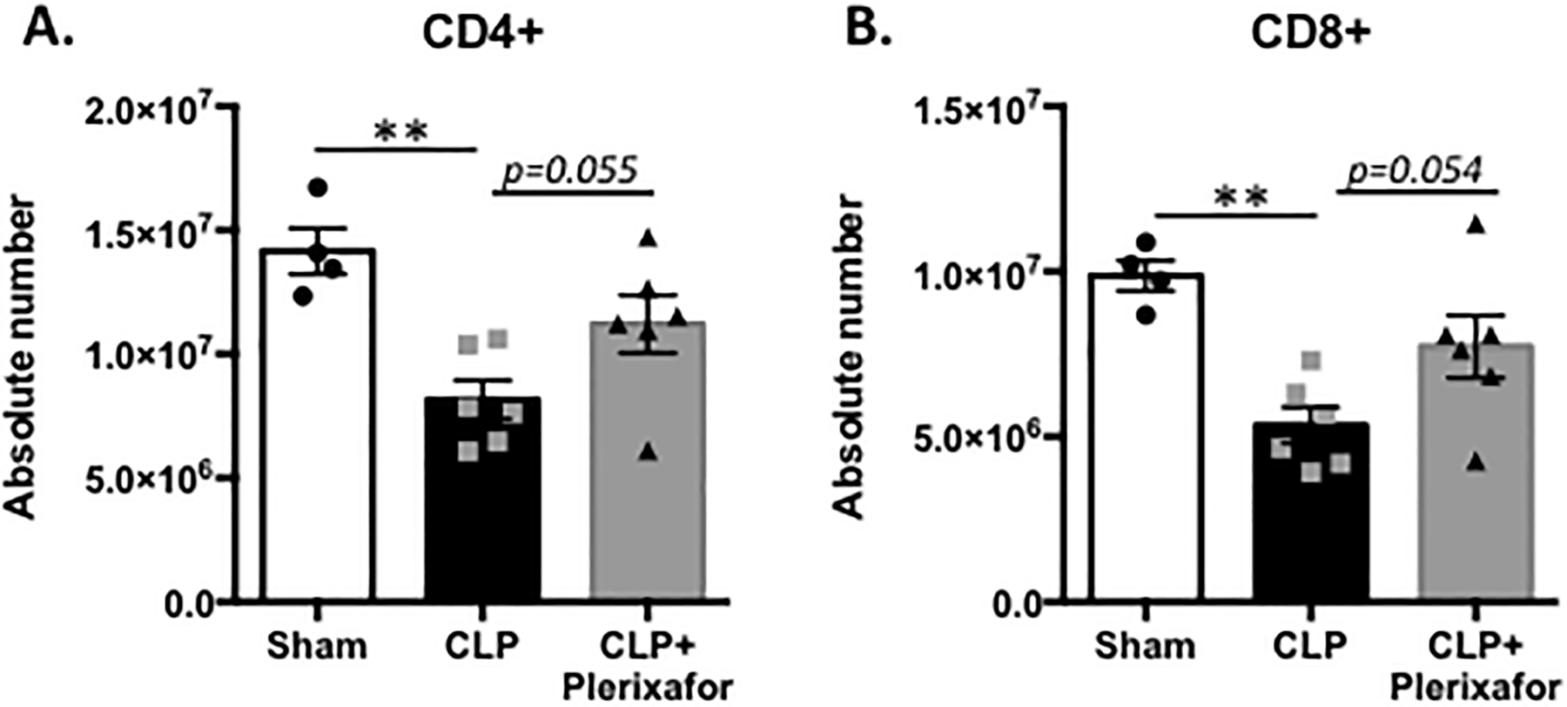
Plerixafor treatment abrogates the loss of peripheral T cells in sepsis. (A) Sepsis results in a decrease in the absolute counts of CD4+ T cells in the spleen at 24 hours post sepsis compared to sham mice (p = 0.0012). Septic mice treated with plerixafor have a trend toward increased absolute counts of CD4+ T cells compared to septic control mice (p = 0.055). (B) Similarly, septic mice have a decrease in the absolute number of circulating CD8+ T cells in the blood compared to sham mice (p = 0.0004) and septic mice treated with plerixafor have a trend toward an increase in circulating CD8+ T cells compared to septic control mice (p = 0.054). n = 4-6/group. Representative of 2 independent experiments with a total of n = 8-10/group.

To determine the effect of CXCR4 blockade on the number of peripheral T cells during sepsis, spleens were harvested at 24h from sham, septic control mice (CLP), and septic mice treated with plerixafor (CLP+Plerixafor), and absolute counts of CD4^+^ T cells and CD8^+^ T cells were analyzed. Sepsis resulted in a decrease in the absolute count of splenic CD4^+^ T cells at 24 hours post sepsis compared to sham mice (p = 0.0012). In contrast, spleens from septic mice treated with plerixafor contained numbers of CD4^+^ T cells that were not significantly different from sham animals, and exhibited a trend toward an increase in the absolute number of CD4^+^ T cells relative to untreated CLP controls (p = 0.055, Fig 3A). Similarly, septic mice exhibited a decrease in the absolute number of splenic CD8^+^ T cells compared to sham mice (p<0.0004), but septic mice treated with plerixafor demonstrated a trend toward an increase in CD8^+^ T cells in the spleen compared to untreated septic animals (p = 0.054, Fig 3B). These effects of plerixafor on peripheral T cell counts were not the result of diminished T cell apoptosis, as frequencies of AnnexinV^+^ 7-AAD^+^ T cells in splenocytes were not different between untreated and plerixafor-treated septic animals (data not shown).

### CXCR4-blockade decreased the percentage of PD-1 expressing T cells during sepsis

**Fig 4:**
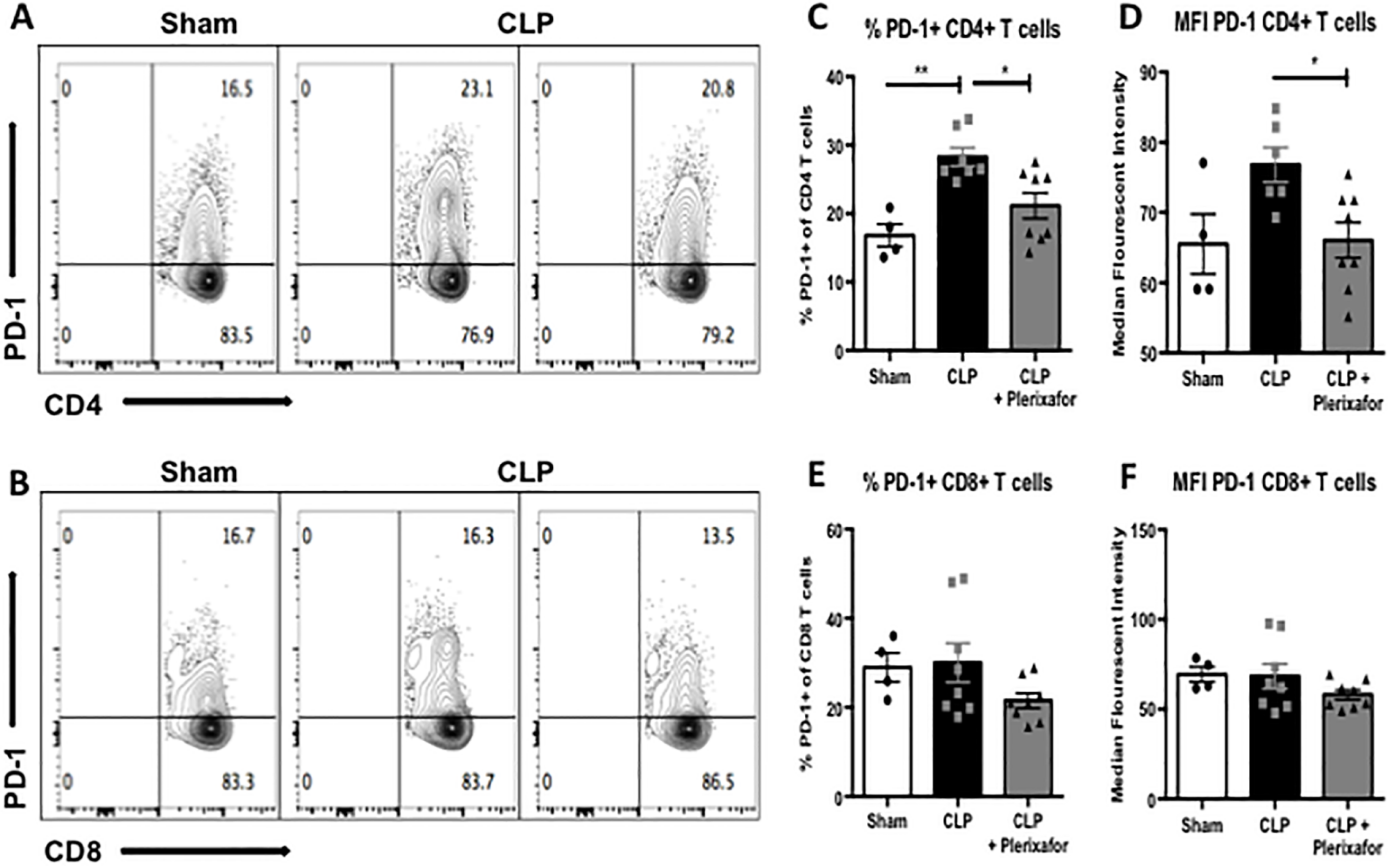
CXCR4 blockade decreased the percentage of PD-1 expressing adaptive immune cells in sepsis. **(A-B)** Representative flow plots (gated on CD3 cells) demonstrating PD-1 expression in CD4^+^ T cells and CD8^+^ T cells. (**C**) The frequency of PD-1^+^ CD4^+^ T cells was significantly increased in septic mice compared to sham mice (28.3% vs. 16.8%; p = 0.002). When septic mice were treated with plerixafor, the frequency of PD-1^+^ CD4^+^ T cells was significantly decreased compared to septic control mice (21.1% vs. 28.3%; p = 0.0156). (**D**) In septic mice treated with plerixafor, the per-cell expression of PD-1 on CD4^+^ T cells, as measured by MFI, was significantly decreased compared to septic control mice (66.1 vs. 76.8; p = 0.033). (**E-F**) There was no statistically significant difference in frequency (**E**) or MFI (**F**) of PD-1^+^ CD8^+^ T cells in septic mice treated with plerixafor compared to septic control mice (21.5% vs. 30.0%; p = 0.169 and 58.0 vs. 68.3; p = 0.327, respectively). N = 4–8 mice/group. Representative of 3 independent experiments with a total of 12–24 mice/group.

Given the results that plerixafor treatment increased the number of peripheral CD4^+^ and CD8^+^ T cells during sepsis, we sought to interrogate the immunophenotypic characteristics of these two cell populations to determine the effect of plerixafor administration on co-inhibitory marker expression, specifically the expression of PD-1. As expected, the frequency of PD-1^+^ CD4^+^ T cells was significantly increased in septic mice as compared to sham mice (Fig 4A and 4C; 28.3% vs. 16.8%; p = 0.002). When septic mice were treated with plerixafor, the frequency of PD-1^+^ cells among CD4^+^ T cells was significantly decreased compared to septic control mice (21.1% vs. 28.3%; p = 0.0156; Fig 4A and 4C). Additionally, in septic mice treated with plerixafor, the level of PD-1 expression on a per-cell basis on CD4^+^ T cells, as measured by MFI (median fluorescence intensity), was significantly decreased as compared to septic control mice (66.1 vs. 76.8; p = 0.033; Fig 4D). Analysis of the effect of plerixafor administration on PD-1 expressing CD8^+^ T cells (Fig 4B) revealed a trend toward a decreased percentage and MFI of PD-1+ among CD8^+^ T cells as compared to septic control mice (Fig 4E and 4F) but these results did not reach statistical significance.

### CXCR4-blockade decreased the percentage of LAG-3 and 2B4 expressing CD4^+^ T cells during sepsis

**Fig 5:**
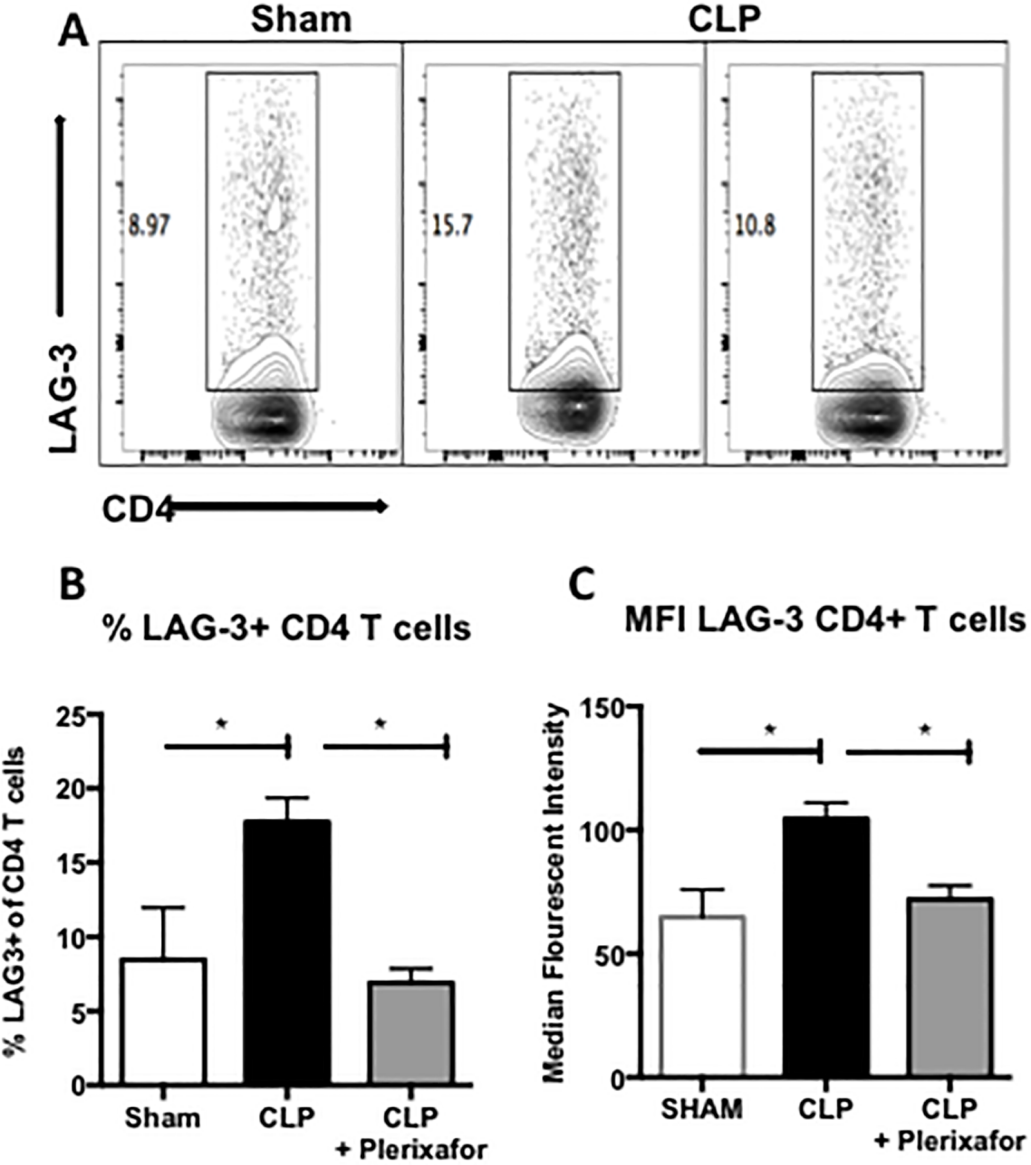
Plerixafor decreased the frequency of LAG-3+ CD4+ T cells in septic mice. (**A**) Representative flow plots (gated on CD3^+^ cells) demonstrating LAG-3 expression on CD4^+^ T cells. **(B)** Septic mice exhibited a significant increase in frequency of LAG-3^+^ CD4^+^ T cells compared to sham mice (17.7% vs. 8.5%; p = 0.046). When septic mice were treated with plerixafor, the frequency of LAG-3^+^ CD4^+^ T cells was significantly decreased compared to septic control mice (6.9% vs. 17.7%; p = 0.0173). (**C**) Septic mice exhibited a significant increase in the per-cell expression of LAG-3 on CD4^+^ T cells compared to sham mice (MFI 104.4 vs. 64.6; p = 0.025) and septic mice treated with plerixafor exhibited a significant decrease in the expression of LAG-3 on CD4^+^ T cells compared to septic control mice (71.9 vs. 104.4; p = 0.0461). N = 3–5 mice/group. Representative of 3 independent experiments with a total of 12 mice/group.

In light of the decrease in PD-1 expressing CD4^+^ T cells observed in septic animals treated with plerixafor, we next evaluated the effect of CXCR4 blockade on the expression of additional coinhibitory markers on CD4^+^ T cells, including LAG-3 and 2B4. As expected, septic mice exhibited a significant increase in the frequency of LAG-3^+^ CD4^+^ T cells compared to sham mice (17.7% vs. 8.5%; p = 0.046; Fig 5A and 5B). When septic mice were treated with plerixafor, the frequency of LAG-3^+^ CD4^+^ T cells was significantly decreased compared to septic control mice (6.9% vs. 17.7%; p = 0.0173; Fig 5A and 5B). Additionally, septic mice demonstrated a significant increase in the expression of LAG-3 on CD4^+^ T cells on a per-cell basis as compared to sham mice, as measured by the MFI (104.4 vs. 64.6; p = 0.025; Fig 5C), and septic mice treated with plerixafor exhibited a significant decrease in the expression of LAG-3 on CD4^+^ T cells as compared to septic control mice (71.9 vs. 104.4; p = 0.0461; Fig 5C).

**Fig 6:**
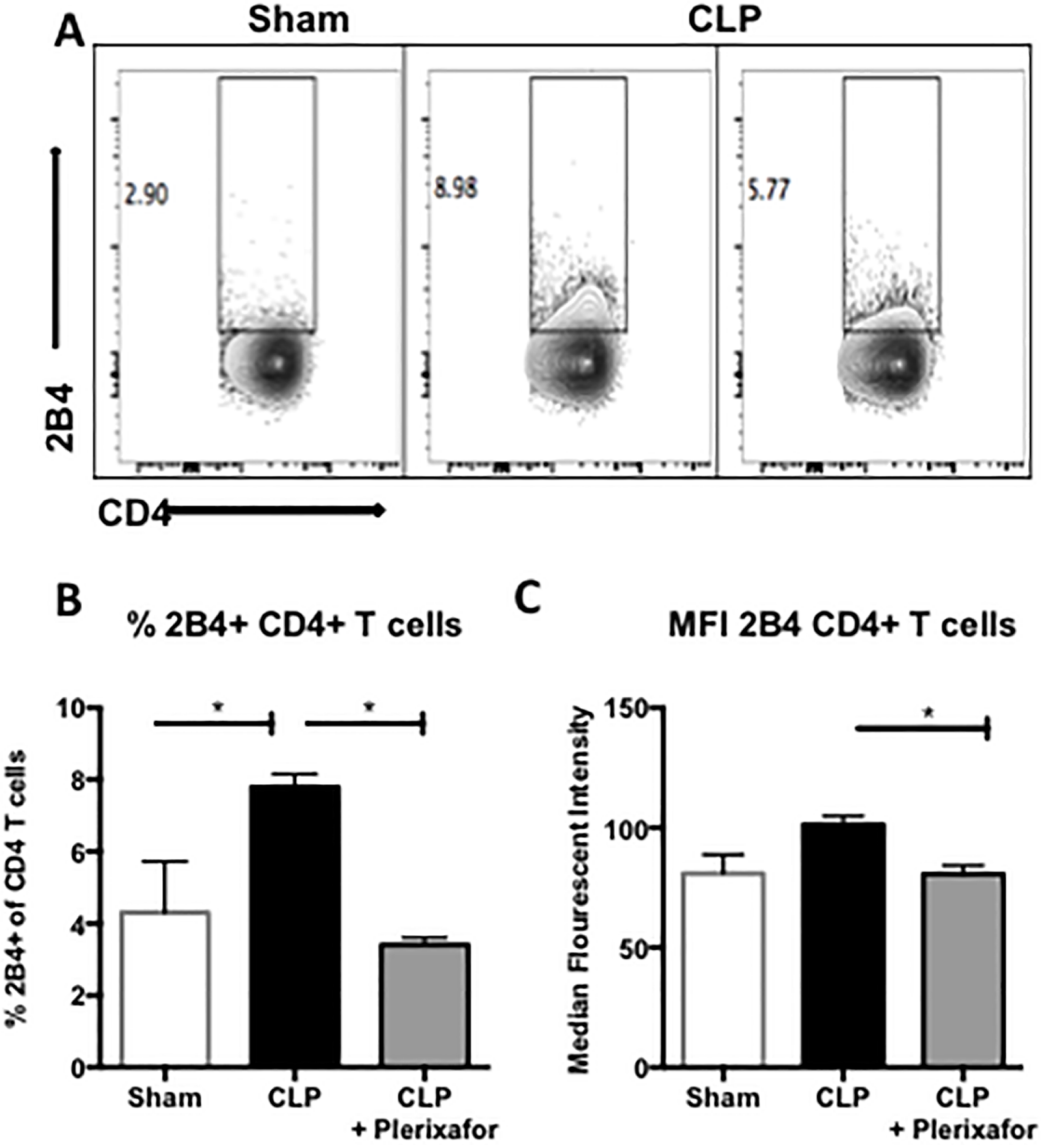
Plerixafor decreased the frequency of 2B4^+^ CD4^+^ T cells in septic mice. **(A**) Representative flow plots (gated on CD3^+^ cells) demonstrating 2B4 expression on CD4^+^ T cells. **(B)** Septic mice exhibited a significant increase in frequency of 2B4^+^ CD4^+^ T cells compared to sham mice (7.79% vs. 4.3%; p = 0.0421). When septic mice were treated with plerixafor, the frequency of 2B4^+^ CD4^+^ T cells was significantly decreased compared to septic control mice (3.4% vs. 7.79%; p = 0.0108). (**C**) Septic control mice exhibited an increase in the per-cell expression of 2B4 on CD4^+^ T cells compared to sham mice, as measured by the MFI (101.4 vs. 80.9; p = 0.068). Septic mice treated with plerixafor exhibited a significant decrease in the expression of 2B4 on CD4^+^ T cells compared to septic control mice (80.6 vs. 101.4; p = 0.05). N = 3–5 mice/group. Representative of 3 independent experiments with a total of 9–15 mice/group.

Examination of the expression of 2B4 on CD4^+^ T cells revealed similar results. Septic mice exhibited a significant increase in frequency of 2B4^+^ CD4^+^ T cells as compared to sham mice (7.79% vs. 4.3%; p = 0.0421; Fig 6A and 6B). However, when septic mice were treated with plerixafor, the frequency of 2B4^+^ CD4^+^ T cells was significantly decreased compared to septic control mice (3.4% vs. 7.79%; p = 0.0108; Fig 6A and 6B). Likewise, septic control mice demonstrated an increase in the per-cell expression of 2B4 on CD4^+^ T cells compared to sham mice, as measured by the MFI (101.4 vs. 80.9; p = 0.068; Fig 6C); septic mice treated with plerixafor displayed a significant decrease in the expression of 2B4 on CD4^+^ T cells compared to untreated septic animals (80.6 vs. 101.4; p = 0.05; Fig 6C).

### Plerixafor treatment did not affect frequencies of cytokine-producing T cells or the levels of circulating pro- or anti-inflammatory cytokines

**Fig 7:**
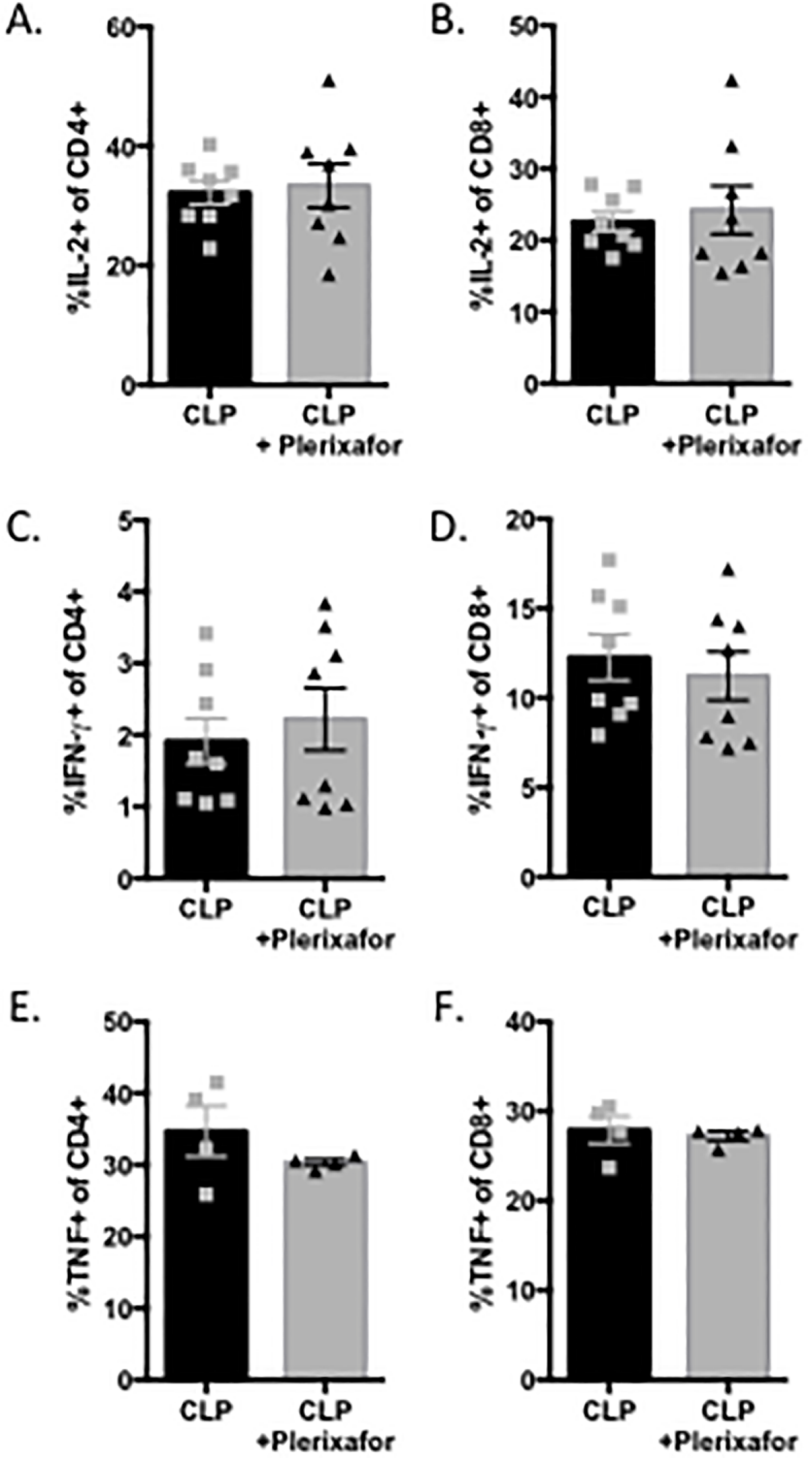
Cytokine production by CD4+ and CD8+ T cells is similar in plerixafor-treated septic animals as compared to control septic animals. Control or plerixafor-treated septic animals were sacrificed at 24h post-CLP and splenocytes were restimulated ex vivo with PMA/ionomycin for 4 h. Cells were fixed, permeabilized, and frequencies of IL-2 (A-B), IFN-g (C-D), and TNF (E-F) secreting CD4+ (A, C, E) and CD8+ (B, D, F) T cells were assessed by flow cytometry. Data shown are cumulative from two independent experiments (n = 4-8/group).

**Fig 8:**
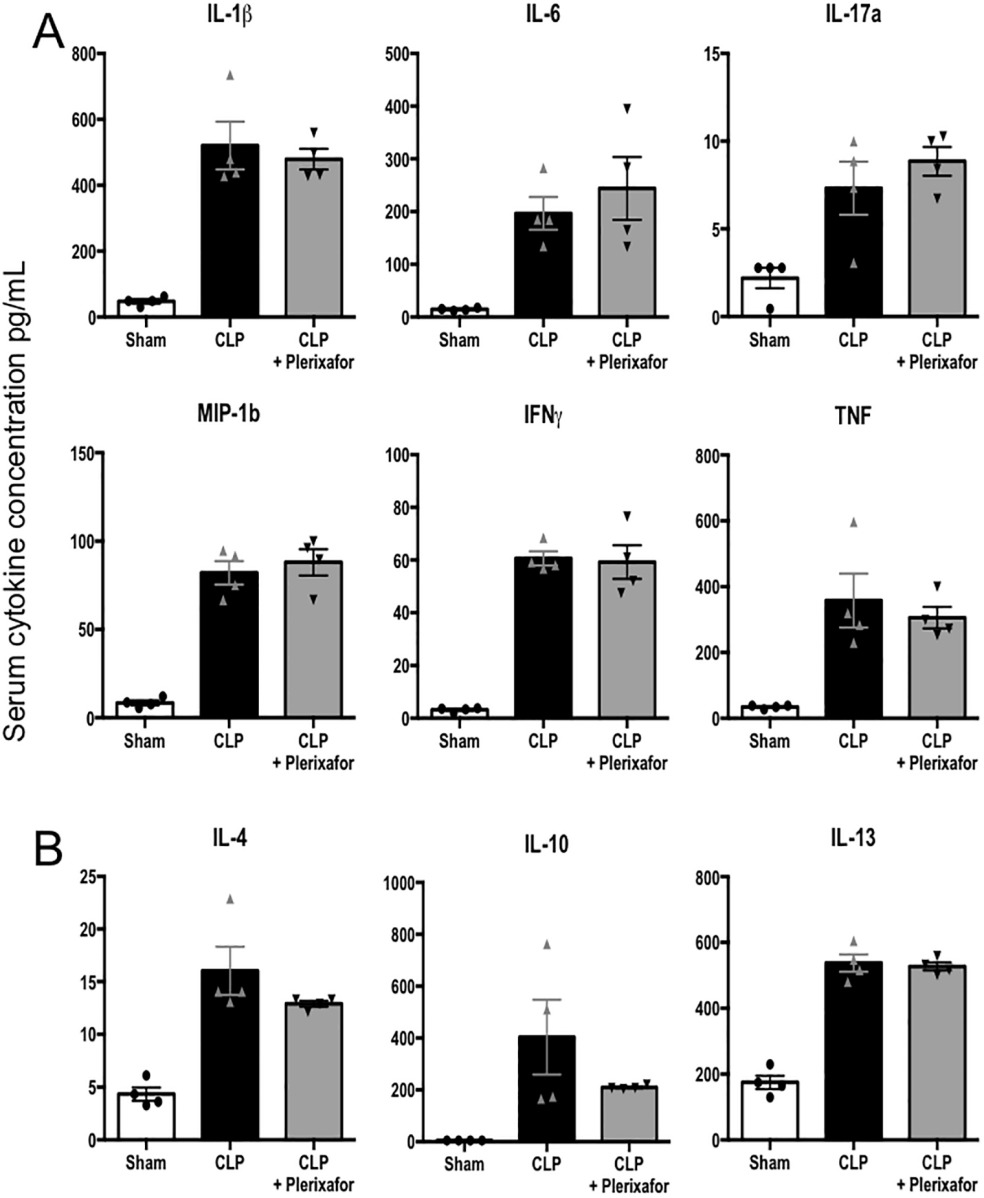
CXCR4 blockade did not affect levels of circulating inflammatory cytokines in sepsis. Multiple pro-inflammatory (**A**) and anti-inflammatory (**B**) cytokine levels were measured in the serum of sham, septic control and plerixafor treated septic mice. No statistically significant differences were observed between septic control mice and septic mice treated with plerixafor. n = 4/group. Representative of 2 independent experiments with a total of 8 mice/group.

To determine the effect of CXCR4 blockade on T cell functionality during sepsis, we harvested splenocytes at 24 hours post-CLP from either untreated mice or mice treated with plerixafor as described above, and restimulated them with PMA/ionomycin for 4 hours in vitro to assess their ability to produce the effector cytokines IL-2, IFN-γ, and TNF. We observed that frequencies of IL-2-, IFN-g, and TNF-secreting both CD4^+^ and CD8^+^ T cells were not different between untreated and plerixafor-treated groups (Fig 7A–7F). Next, to determine the effect of CXCR4 antagonism on the systemic cytokine environment during sepsis, we studied the levels of multiple circulating pro-inflammatory (Fig 8A) and anti-inflammatory cytokines (Fig 8B) known to be elevated in sepsis in sham controls, CLP animals, and CLP animals treated with plerixafor. While all cytokines analyzed were significantly higher in both septic groups as compared to sham controls, no statistically significant differences in levels of cytokines between untreated CLP mice and and plerixafor-treated CLP animals at 24 hours post-surgery were identified, although there was a trend toward decreased IL-4 and IL-10 in plerixafor-treated septic animals.

## Discussion

In this series of investigations, we found that CXCR4 blockade improved survival in murine polymicrobial sepsis, increased the absolute number of circulating CD4^+^ and CD8^+^ T cells, and mitigated sepsis-induced T cell exhaustion phenotypes. Specifically, plerixafor treatment following CLP significantly decreased the percentage of PD-1, LAG-3, and 2B4-expressing CD4^+^ T cells in septic mice. Given these findings, one potential explanation for the improved survival observed following CXCR4 blockade is that mobilizing bone marrow stores of functional immune cells replenishes circulating populations of T cells that have been diminished in the periphery due to sepsis-induced apoptosis or migration to the site of inflammation. This hypothesis is supported by our findings of increased numbers of CD4^+^ and CD8^+^ T cells in the periphery of plerixafor-treated septic mice as compared to control septic mice, and is consistent with the known mechanism of action of the drug [33]. Recruitment of “fresh” T cell populations from bone marrow stores may replenish splenic T cell populations with cells that have been less affected by the inflammatory milieu of the circulation. As such, we speculate that plerixafor treatment results in a decrease in PD-1^+^ cells not because of a down-regulation in PD-1 on existing PD-1^hi^ cells, but because the population of PD-1^hi^ cells may be “diluted out” by the influx of non-exhausted T cells mobilized from the bone marrow, thereby mitigating T cell exhaustion and immune-incompetence that occurs during sepsis.

Alternatively, CXCR4 antagonism could be having a direct effect on T cell activation and/or expansion independently of any effect of bone marrow niche mobilization. Indeed, CXCR4 has been shown to associate with the TCR complex, and CXCL12/CXCR4 signaling is necessary for TCR-initiated immune synapse formation, enhanced phosphorylation of early signaling molecules, and thymic β selection [34–39]. A recent study also showed that upon ligation of TCR, the TCR associates with and trans-activates CXCR4 in order to activate a PREX1-Rac1 signaling pathway that stabilizes IL-2, IL-4, and IL-10 mRNA transcripts [40]. Thus, it is possible that increased CXCR4 expression during sepsis may amplify this pathway and result in enhanced IL-2, IL-4, and/or IL-10 secretion. Consistent with this possibility, our data indicated that CXCR4 antagonism with plerixafor resulted in a trend toward reduced serum concentrations of IL-4 and IL-10 in septic animals (Fig 7).

As discussed above, our study shows that CXCR4 is upregulated on naïve CD4^+^ and CD8^+^ T cells, as well as CD4^+^ T_CM_, during sepsis. Interestingly, two pathways of CXCR4 upregulation on T cells have been described. One is mediated via ligation of common gamma chain cytokine receptors including IL-2, IL-4, IL-7, IL-15, and IL-21 [41], which are not the classic inflammatory cytokines thought to be induced following CLP. An alternative pathway of CXCR4 upregulation has also been described, driven by NFkB binding to the *Cxcr4* promoter [42]. This pathway depends on CD154/CD40 and CD134/CD134L interactions, and is independent of TCR ligation [43]. Intriguingly, CD40/CD134-dependent CXCR4 ligation was actually inhibited in the presence of CD3-mediated signals. Taken together with our results, these data suggest that bystander activation of T cells in an inflammatory environment in which CD40 and/or CD134 signals are present in the absence of cognate antigen may drive CXCR4 upregulation during sepsis.

Moreover, the effect of sepsis on CXCR4 expression was T cell subset-specific, in that expression was not upregulated on CD8^+^ T_CM_, or on T_EM_ in either the CD4^+^ or CD8^+^ compartments. Our data showing that CXCR4 was most highly upregulated on naïve and T_CM_ cells following sepsis are consistent with findings from human studies which show that the highest expression of CXCR4 is on naïve T cells, with minimal expression on highly differentiated T_EM_ or effector memory-RA (TEMRA) cells [44]. Given what is known about the mechanisms that control CXCR4 expression on T cells, it is possible that this differential expression results from differential expression of common gamma chain cytokine receptors, TNF family members CD40 and/or CD134, or epigenetic remodeling of the *Cxcr4* locus in more highly differentiated T cell subsets that render the promoter inaccessible to NFkB.

Our results indicate a strong survival advantage in animals treated with the CXCR4 antagonist plerixafor. In support of this, a recent study of human septic patients revealed that CXCL12 levels were higher in patients with severe sepsis/septic shock as compared to healthy subjects. Moreover, the same study also found that patients who survived their septic insult possessed lower serum levels of CXCL12 than those who died [26]. The data from our CLP model together with these clinical findings therefore lead us to conclude that engagement of the CXCR4/CXCL12 pathway is deleterious during sepsis. These results are seemingly inconsistent with earlier findings from Efron’s group, which showed that blockade of the CXCR4 ligand CXCL12 resulted in increased mortality in murine models of CLP and *Pseudomonas* infection [45]. The finding that blocking the receptor (CXCR4) yields a result distinct from that observed when blocking the ligand (CXCL12) may suggest that one or more alternate binding partners is available and capable of modulating the response during sepsis. Adding to the complexity, administration of plerixafor in a model of LPS-induced endotoxemia resulted in enhanced serum levels of TNF, IFN-γ and NO levels and overall worsened outcomes [46]. These results highlight differences between models of LPS-induced endotoxemia and septic shock induced by a live, replicating organism.

Our study is limited by the fact that the data presented here represent only a single time point post- sepsis, and by the focus on analyses of the T cell compartment in plerixafor-treated animals. CXCR4 is expressed on many immune cell types including monocytes and DC, and our preliminary data suggest that CXCR4 may also be upregulated on these lineages during CLP (data not shown). Thus, identification of the effects of plerixafor on the T cell compartment during sepsis does not preclude the possibility that CXCR4 antagonism could affect other immune cell types during sepsis. Additional analyses are warranted in this regard. In sum, our study identifies an important pathway that modulates immune dysfunction and mortality following sepsis, which may hold promise as a target for future therapeutic intervention in septic patients.

## References

[1] Gaieski DF, Edwards JM, Kallan MJ, Carr BG. Benchmarking the incidence and mortality of severe sepsis in the United States. Crit Care Med. 2013;41(5):1167–74. Epub 2013/02/28. doi: 10.1097/CCM.0b013e31827c09f8 . 23442987 10.1097/CCM.0b013e31827c09f8

[2] Singer M, Deutschman CS, Seymour CW, Shankar-Hari M, Annane D, Bauer M, et al The Third International Consensus Definitions for Sepsis and Septic Shock (Sepsis-3). JAMA. 2016;315(8):801–10. doi: 10.1001/jama.2016.0287 26903338 10.1001/jama.2016.0287 PMC4968574

[3] Dellinger RP, Levy MM, Rhodes A, Annane D, Gerlach H, Opal SM, et al Surviving Sepsis Campaign: international guidelines for management of severe sepsis and septic shock, 2012. Intensive Care Med. 2013;39(2):165–228. doi: 10.1007/s00134-012-2769-8 . 23361625 10.1007/s00134-012-2769-8 PMC7095153

[4] Pro CI, Yealy DM, Kellum JA, Huang DT, Barnato AE, Weissfeld LA, et al A randomized trial of protocol-based care for early septic shock. N Engl J Med. 2014;370(18):1683–93. doi: 10.1056/NEJMoa1401602 24635773 10.1056/NEJMoa1401602 PMC4101700

[5] Hotchkiss RS, Monneret G, Payen D. Sepsis-induced immunosuppression: from cellular dysfunctions to immunotherapy. Nat Rev Immunol. 2013;13(12):862–74. doi: 10.1038/nri3552 . 24232462 10.1038/nri3552 PMC4077177

[6] Hotchkiss RS, Monneret G, Payen D. Immunosuppression in sepsis: a novel understanding of the disorder and a new therapeutic approach. Lancet Infect Dis. 2013;13(3):260–8. doi: 10.1016/S1473-3099(13)70001-X 23427891 10.1016/S1473-3099(13)70001-X PMC3798159

[7] Payen D, Monneret G, Hotchkiss R. Immunotherapy—a potential new way forward in the treatment of sepsis. Crit Care. 2013;17(1):118 doi: 10.1186/cc12490 23425441 10.1186/cc12490 PMC4056021

[8] Boomer JS, Green JM, Hotchkiss RS. The changing immune system in sepsis: is individualized immuno-modulatory therapy the answer? Virulence. 2014;5(1):45–56. doi: 10.4161/viru.26516 24067565 10.4161/viru.26516 PMC3916383

[9] Leentjens J, Kox M, van der Hoeven JG, Netea MG, Pickkers P. Immunotherapy for the adjunctive treatment of sepsis: from immunosuppression to immunostimulation. Time for a paradigm change? Am J Respir Crit Care Med. 2013;187(12):1287–93. doi: 10.1164/rccm.201301-0036CP . 23590272 10.1164/rccm.201301-0036CP

[10] Fife BT, Bluestone JA. Control of peripheral T-cell tolerance and autoimmunity via the CTLA-4 and PD-1 pathways. Immunol Rev. 2008;224:166–82. doi: 10.1111/j.1600-065X.2008.00662.x . 18759926 10.1111/j.1600-065X.2008.00662.x

[11] Keir ME, Butte MJ, Freeman GJ, Sharpe AH. PD-1 and its ligands in tolerance and immunity. Annu Rev Immunol. 2008;26:677–704. doi: 10.1146/annurev.immunol.26.021607.090331 . 18173375 10.1146/annurev.immunol.26.021607.090331 PMC10637733

[12] Huang X, Venet F, Wang YL, Lepape A, Yuan Z, Chen Y, et al PD-1 expression by macrophages plays a pathologic role in altering microbial clearance and the innate inflammatory response to sepsis. Proc Natl Acad Sci U S A. 2009;106(15):6303–8. doi: 10.1073/pnas.0809422106 19332785 10.1073/pnas.0809422106 PMC2669369

[13] Shubin NJ, Chung CS, Heffernan DS, Irwin LR, Monaghan SF, Ayala A. BTLA expression contributes to septic morbidity and mortality by inducing innate inflammatory cell dysfunction. J Leukoc Biol. 2012;92(3):593–603. Epub 2012/03/31. doi: 10.1189/jlb.1211641 22459947 10.1189/jlb.1211641 PMC3427605

[14] Shubin NJ, Monaghan SF, Heffernan DS, Chung CS, Ayala A. B and T lymphocyte attenuator expression on CD4+ T-cells associates with sepsis and subsequent infections in ICU patients. Crit Care. 2013;17(6):R276 Epub 2013/12/03. doi: 10.1186/cc13131 . 24289156 10.1186/cc13131 PMC4057112

[15] Chang KC, Burnham CA, Compton SM, Rasche DP, Mazuski RJ, McDonough JS, et al Blockade of the negative co-stimulatory molecules PD-1 and CTLA-4 improves survival in primary and secondary fungal sepsis. Crit Care. 2013;17(3):R85 doi: 10.1186/cc12711 23663657 10.1186/cc12711 PMC3706819

[16] Chen CW, Mittal R, Klingensmith NJ, Burd EM, Terhorst C, Martin GS, et al Cutting Edge: 2B4-Mediated Coinhibition of CD4+ T Cells Underlies Mortality in Experimental Sepsis. J Immunol. 2017 doi: 10.4049/jimmunol.1700375 . 28768726 10.4049/jimmunol.1700375 PMC5587400

[17] Venet F, Foray AP, Villars-Mechin A, Malcus C, Poitevin-Later F, Lepape A, et al IL-7 restores lymphocyte functions in septic patients. J Immunol. 2012;189(10):5073–81. doi: 10.4049/jimmunol.1202062 . 23053510 10.4049/jimmunol.1202062

[18] Guignant C, Lepape A, Huang X, Kherouf H, Denis L, Poitevin F, et al Programmed death-1 levels correlate with increased mortality, nosocomial infection and immune dysfunctions in septic shock patients. Crit Care. 2011;15(2):R99 Epub 2011/03/23. doi: 10.1186/cc10112 21418617 10.1186/cc10112 PMC3219369

[19] Brahmamdam P, Inoue S, Unsinger J, Chang KC, McDunn JE, Hotchkiss RS. Delayed administration of anti-PD-1 antibody reverses immune dysfunction and improves survival during sepsis. J Leukoc Biol. 2010;88(2):233–40. doi: 10.1189/jlb.0110037 . 20483923 10.1189/jlb.0110037 PMC6607999

[20] Drewry AM, Samra N, Skrupky LP, Fuller BM, Compton SM, Hotchkiss RS. Persistent lymphopenia after diagnosis of sepsis predicts mortality. Shock. 2014;42(5):383–91. doi: 10.1097/SHK.0000000000000234 . 25051284 10.1097/SHK.0000000000000234 PMC4362626

[21] Nie Y, Waite J, Brewer F, Sunshine MJ, Littman DR, Zou YR. The role of CXCR4 in maintaining peripheral B cell compartments and humoral immunity. J Exp Med. 2004;200(9):1145–56. doi: 10.1084/jem.20041185 15520246 10.1084/jem.20041185 PMC2211858

[22] Eash KJ, Greenbaum AM, Gopalan PK, Link DC. CXCR2 and CXCR4 antagonistically regulate neutrophil trafficking from murine bone marrow. J Clin Invest. 2010;120(7):2423–31. doi: 10.1172/JCI41649 20516641 10.1172/JCI41649 PMC2898597

[23] Kean LS, Sen S, Onabajo O, Singh K, Robertson J, Stempora L, et al Significant mobilization of both conventional and regulatory T cells with AMD3100. Blood. 2011;118(25):6580–90. doi: 10.1182/blood-2011-06-359331 21989987 10.1182/blood-2011-06-359331 PMC3242720

[24] Kohara H, Omatsu Y, Sugiyama T, Noda M, Fujii N, Nagasawa T. Development of plasmacytoid dendritic cells in bone marrow stromal cell niches requires CXCL12-CXCR4 chemokine signaling. Blood. 2007;110(13):4153–60. doi: 10.1182/blood-2007-04-084210 . 17827391 10.1182/blood-2007-04-084210

[25] Liu Q, Li Z, Gao JL, Wan W, Ganesan S, McDermott DH, et al CXCR4 antagonist AMD3100 redistributes leukocytes from primary immune organs to secondary immune organs, lung, and blood in mice. Eur J Immunol. 2015;45(6):1855–67. doi: 10.1002/eji.201445245 25801950 10.1002/eji.201445245 PMC4461468

[26] Franchini S, Marciano T, Sorlini C, Campochiaro C, Tresoldi M, Sabbadini MG, et al Serum CXCL12 levels on hospital admission predict mortality in patients with severe sepsis/septic shock. The American journal of emergency medicine. 2015;33(12):1802–4. doi: 10.1016/j.ajem.2015.08.047 . 26387470 10.1016/j.ajem.2015.08.047

[27] Fruehauf S. Current clinical indications for plerixafor. Transfus Med Hemother. 2013;40(4):246–50. doi: 10.1159/000354229 24415962 10.1159/000354229 PMC3776405

[28] Hummel S, Van Aken H, Zarbock A. Inhibitors of CXC chemokine receptor type 4: putative therapeutic approaches in inflammatory diseases. Curr Opin Hematol. 2014;21(1):29–36. . 24275689 10.1097/MOH.0000000000000002

[29] Matthys P, Hatse S, Vermeire K, Wuyts A, Bridger G, Henson GW, et al AMD3100, a potent and specific antagonist of the stromal cell-derived factor-1 chemokine receptor CXCR4, inhibits autoimmune joint inflammation in IFN-gamma receptor-deficient mice. J Immunol. 2001;167(8):4686–92. . 11591799 10.4049/jimmunol.167.8.4686

[30] Xia XM, Wang FY, Zhou J, Hu KF, Li SW, Zou BB. CXCR4 antagonist AMD3100 modulates claudin expression and intestinal barrier function in experimental colitis. PLoS One. 2011;6(11):e27282 doi: 10.1371/journal.pone.0027282 22073304 10.1371/journal.pone.0027282 PMC3207859

[31] Coopersmith CM, Chang KC, Swanson PE, Tinsley KW, Stromberg PE, Buchman TG, et al Overexpression of Bcl-2 in the intestinal epithelium improves survival in septic mice. Crit Care Med. 2002;30(1):195–201. . 11902262 10.1097/00003246-200201000-00028

[32] Gilson CR, Milas Z, Gangappa S, Hollenbaugh D, Pearson TC, Ford ML, et al Anti-CD40 monoclonal antibody synergizes with CTLA4-Ig in promoting long-term graft survival in murine models of transplantation. J Immunol. 2009;183(3):1625–35. doi: 10.4049/jimmunol.0900339 19592649 10.4049/jimmunol.0900339 PMC2828346

[33] De Clercq E. AMD3100/CXCR4 Inhibitor. Front Immunol. 2015;6:276 doi: 10.3389/fimmu.2015.00276 26106388 10.3389/fimmu.2015.00276 PMC4459229

[34] Golec DP, Dower NA, Stone JC, Baldwin TA. RasGRP1, but not RasGRP3, is required for efficient thymic beta-selection and ERK activation downstream of CXCR4. PLoS One. 2013;8(1):e53300 doi: 10.1371/journal.pone.0053300 23308188 10.1371/journal.pone.0053300 PMC3538756

[35] Hernandez-Lopez C, Valencia J, Hidalgo L, Martinez VG, Zapata AG, Sacedon R, et al CXCL12/CXCR4 signaling promotes human thymic dendritic cell survival regulating the Bcl-2/Bax ratio. Immunol Lett. 2008;120(1–2):72–8. doi: 10.1016/j.imlet.2008.07.006 . 18692524 10.1016/j.imlet.2008.07.006

[36] Janas ML, Turner M. Stromal cell-derived factor 1alpha and CXCR4: newly defined requirements for efficient thymic beta-selection. Trends Immunol. 2010;31(10):370–6. doi: 10.1016/j.it.2010.07.002 . 20829112 10.1016/j.it.2010.07.002

[37] Janas ML, Varano G, Gudmundsson K, Noda M, Nagasawa T, Turner M. Thymic development beyond beta-selection requires phosphatidylinositol 3-kinase activation by CXCR4. J Exp Med. 2010;207(1):247–61. doi: 10.1084/jem.20091430 20038597 10.1084/jem.20091430 PMC2812547

[38] Trampont PC, Tosello-Trampont AC, Shen Y, Duley AK, Sutherland AE, Bender TP, et al CXCR4 acts as a costimulator during thymic beta-selection. Nat Immunol. 2010;11(2):162–70. doi: 10.1038/ni.1830 20010845 10.1038/ni.1830 PMC2808461

[39] Smith X, Schneider H, Kohler K, Liu H, Lu Y, Rudd CE. The chemokine CXCL12 generates costimulatory signals in T cells to enhance phosphorylation and clustering of the adaptor protein SLP-76. Sci Signal. 2013;6(286):ra65 doi: 10.1126/scisignal.2004018 . 23901140 10.1126/scisignal.2004018

[40] Kremer KN, Dinkel BA, Sterner RM, Osborne DG, Jevremovic D, Hedin KE. TCR-CXCR4 signaling stabilizes cytokine mRNA transcripts via a PREX1-Rac1 pathway: implications for CTCL. Blood. 2017 doi: 10.1182/blood-2017-03-770982 . 28694325 10.1182/blood-2017-03-770982 PMC5570680

[41] Nagafuchi Y, Shoda H, Sumitomo S, Nakachi S, Kato R, Tsuchida Y, et al Immunophenotyping of rheumatoid arthritis reveals a linkage between HLA-DRB1 genotype, CXCR4 expression on memory CD4(+) T cells, and disease activity. Scientific reports. 2016;6:29338 doi: 10.1038/srep29338 27385284 10.1038/srep29338 PMC4935954

[42] Arieta Kuksin C, Gonzalez-Perez G, Minter LM. CXCR4 expression on pathogenic T cells facilitates their bone marrow infiltration in a mouse model of aplastic anemia. Blood. 2015;125(13):2087–94. doi: 10.1182/blood-2014-08-594796 25647836 10.1182/blood-2014-08-594796 PMC4375106

[43] Jourdan P, Vendrell JP, Huguet MF, Segondy M, Bousquet J, Pene J, et al Cytokines and cell surface molecules independently induce CXCR4 expression on CD4+ CCR7+ human memory T cells. J Immunol. 2000;165(2):716–24. . 10878344 10.4049/jimmunol.165.2.716

[44] Kobayashi N, Takata H, Yokota S, Takiguchi M. Down-regulation of CXCR4 expression on human CD8+ T cells during peripheral differentiation. Eur J Immunol. 2004;34(12):3370–8. doi: 10.1002/eji.200425587 . 15549771 10.1002/eji.200425587

[45] Delano MJ, Kelly-Scumpia KM, Thayer TC, Winfield RD, Scumpia PO, Cuenca AG, et al Neutrophil mobilization from the bone marrow during polymicrobial sepsis is dependent on CXCL12 signaling. J Immunol. 2011;187(2):911–8. doi: 10.4049/jimmunol.1100588 21690321 10.4049/jimmunol.1100588 PMC3635667

[46] Seemann S, Lupp A. Administration of AMD3100 in endotoxemia is associated with pro-inflammatory, pro-oxidative, and pro-apoptotic effects in vivo. J Biomed Sci. 2016;23(1):68 doi: 10.1186/s12929-016-0286-8 27716214 10.1186/s12929-016-0286-8 PMC5048674

